# Transcriptomic changes induced by applications of a commercial extract of *Ascophyllum nodosum* on tomato plants

**DOI:** 10.1038/s41598-022-11263-z

**Published:** 2022-05-16

**Authors:** Omar Ali, Adesh Ramsubhag, Stephen Daniram Benn Jr. Ramnarine, Jayaraj Jayaraman

**Affiliations:** Department of Life Sciences, Faculty of Science and Technology, The University of the West Indies, St. Augustine, Trinidad and Tobago

**Keywords:** Biotechnology, Molecular biology, Plant sciences

## Abstract

Extracts of *Ascophyllum nodosum* are commonly used as commercial biostimulants in crop production. To further understand the seaweed extract-induced phenomena in plants, a transcriptomic study was conducted. RNA-seq differential gene expression analysis of tomato plants treated with a commercial *A. nodosum* extract formulation (Stimplex) revealed the up-regulation of 635 and down-regulation of 456 genes. Ontology enrichment analysis showed three gene categories were augmented, including biological processes, cellular components, and molecular functions. KEGG pathway analysis revealed that the extract had a strong influence on the expression of genes involved in carbon fixation, secondary metabolism, MAPK-signalling, plant hormone signal transduction, glutathione metabolism, phenylpropanoid and stilbenoid metabolism, and plant-pathogen interactions. qRT-PCR validation analysis using 15 genes established a strong correlation with the RNA sequencing results. The activities of defence enzymes were also significantly enhanced by seaweed extract treatment. Furthermore, AN-SWE treated tomato plants had significantly higher chlorophyll and growth hormone content and showed improved plant growth parameters and nutrient profiles than the control. It is postulated that seaweed extract-induced gene regulation was responsible for favourable plant responses that enabled better growth and tolerance to stress conditions. This study provides evidence at the transcriptomic level for the positive effects of foliar application of the *Ascophyllum nodosum* extract (Stimplex) observed in treated tomato plants.

## Introduction

Biostimulants are any substances or microorganisms, independent of its nutritional content, administered to plants with the goal of improving nutrient use, stress tolerance, and/or crop quality attributes^1^. Seaweed extracts are part of this biostimulant category with high rankings because of its multiple beneficial effects^2,3^. Apart from seaweed-extract based biostimulants, there are others that include humic substances, chemical elements, derivatives of chitosan and chitin, anti-transpirants, inorganic salts, organic materials, free amino acids and other N-containing substances and microorganisms^1^. Numerous reports on seaweed extract-based biostimulants to date show multiple positive effects on plant growth, productivity, and quality measures. These include plant disease suppression, pest reduction, increased plant vigor, increased marketable yield, increased shelf-life and produce quality and even abiotic stresses resistance including drought, salinity and cold^3,4^. These positive responses have been documented over several crop plants such as tomato, sweet pepper, strawberry, cucumber and carrots to name a few^5^, and most of these reports were based on various types of commercial extracts of *Ascophyllum nodosum*.

*Ascophyllum nodosum* is a brown seaweed that is commonly used in the production of biostimulants. Consequently, *A. nodosum* seaweed extract (AN-SWE) is one of the most extensively used and studied seaweed-based biostimulants in the market today because of its chronicle of positive results in the enhancement of crop productivity^6,7^. A variety of methods are utilized to prepare seaweed extracts (SWE), with most resulting in a liquid homogenate containing algal cell components^8^. The use of SWE have now become an integral component of sustainable agriculture and its applicability is predicted to rise dramatically in the near future due to its compatibility with production systems, its organic nature and wide-ranging positive effects^3,5,9,10^. Furthermore, with a wide range of factors such as changing climates and increasing occurrences of pathogen resistance to chemical pesticides, as well as environmental pollution, the significance of using organic biostimulants in sustainable agriculture is becoming paramount. To support the popular usage of Stimplex (a commercial formulation from *A. nodosum* extract) and unravel its fullest potential, a better understanding of the mechanisms of action and its interaction with plant, environment and the crop biosphere is essential. Additionally, in certain countries such as the USA and Mexico, Stimplex is categorized as a biopesticide. However, in the Caribbean territories such as Trinidad and Tobago, Stimplex is not classified as such, which allows its use as a bio-input. This mandates the need for complete validation of its biological effects and the molecular mechanisms it can activate in the plants. Currently, beneficial impacts on agro-economic plant parameters are widely reported, but the basic molecular phenomena have not been thoroughly studied in crops, which leaves large knowledge gaps in this field. The basic knowledge of these tenets is imperative for developing efficient and novel formulations of seaweed extract-based biostimulants.

Transcriptomic analysis is a useful, and powerful method for detecting differences in gene expression in specific tissues, different phases of development, or in response to stimuli. To date, transcriptome investigations of biostimulant effects on plants have revealed comprehensive alterations in gene transcription across numerous processes, pathways and functions of the cell^11^. This type of transcriptome research has shown sensitivity and specificity in identifying differences across biostimulant treatments that cause various plant responses^12^. However, to date, no transcriptomic studies has been reported in tomato plants foliar-treated with an *A. nodosum* extract (Stimplex) as means to trigger defense and growth responses before any type of stress is introduced or encountered. Only a handful of reports showed that some key growth and defence signalling marker genes were up-regulated by seaweed extracts (SWE) through qRT-PCR targeted assays. For instance, the extracts of *A. nodosum, Sargassum vulgare* and *Acanthophora spicifera,* were able to up-regulate *PinII* (jasmonic acid), *Etr1* (ethylene), *PR-1a* (salicylic acid)-mediated signalling, auxin (*IAA*), gibberellin (*Ga2Ox*) and cytokinin (*IPT*) biosynthesis-related genes in tomato and sweet pepper^5,13,14^. This increase in expression was also accompanied by a rise in defense enzyme activities, such as phenylalanine ammonia lyase (PAL), peroxidase (POD), polyphenol oxidase (PPO), chitinase (CHI), and β-1,3 glucanase (GLU), due to pre-treatment with extracts^13,15,16^. These studies are the basis for further investigations into changes in the global gene expression profile of plants treated with Stimplex - *A. nodosum* SWE.

In the current study, the next-generation RNA sequencing technology was employed to investigate global transcriptomic changes in tomato, as a model crop plant system, when primed with an *A. nodosum* extract formulation. The study investigated the differential expression of key genes which are responsible for defence and growth processes following the application of a commercially available *A. nodosum* extract (Stimplex) in tomato.

## Results

### Summary of RNA-sequencing read statistics and DEG report

**Figure 1:**
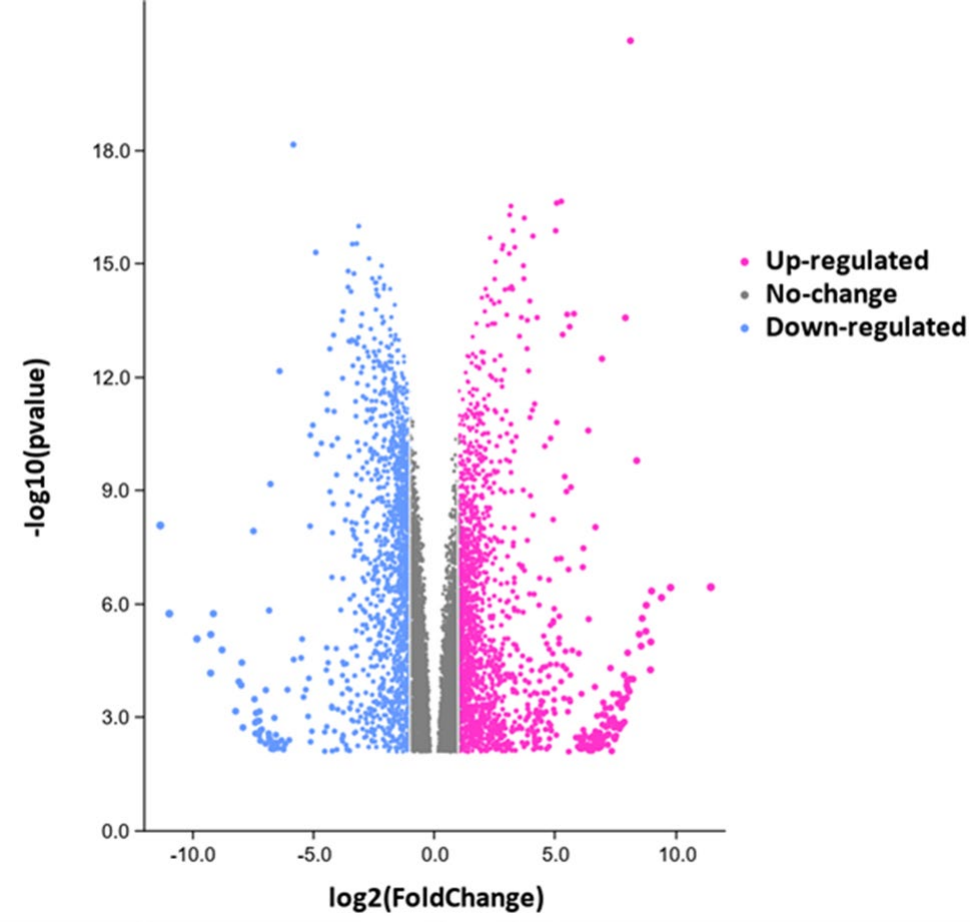
Volcano-plot showing significantly different differentially expressed genes in tomato plants treated with AN-SWE. Genes were considered significantly up-regulated or down-regulated if the corresponding transcript had a log FC of ≥ + 2 and ≤ − 2 respectively and a corrected p-value and FDR of < 0.05.

More than 90% of the reads were retained after quality trimming for all samples. The mapping rate to the reference genome was > 95% for all samples using the HISAT2 algorithm. The volcano plot (Fig. 1) illustrated the general DEG profile for the RNA-sequencing analysis. A log FC of ≥  + 2 and ≤ − 2 was used for up-regulated and down-regulated respectively with a corrected p-value of < 0.05 to indicate expressed transcripts that were significantly different. There were 635 and 456 gene transcripts significantly up-regulated and down-regulated respectively, observed from the RNA-seq data. The multi-dimensional-scaling plot (Supple Fig. S2) showed tight clustering of duplicates for both AN-SWE as well as control samples, indicating overall low variance within the replicates. Furthermore, most of the variance (> 95%) in both treatment groups was explained in dimension one.

### Gene ontology and KEGG pathway enrichment

**Figure 2:**
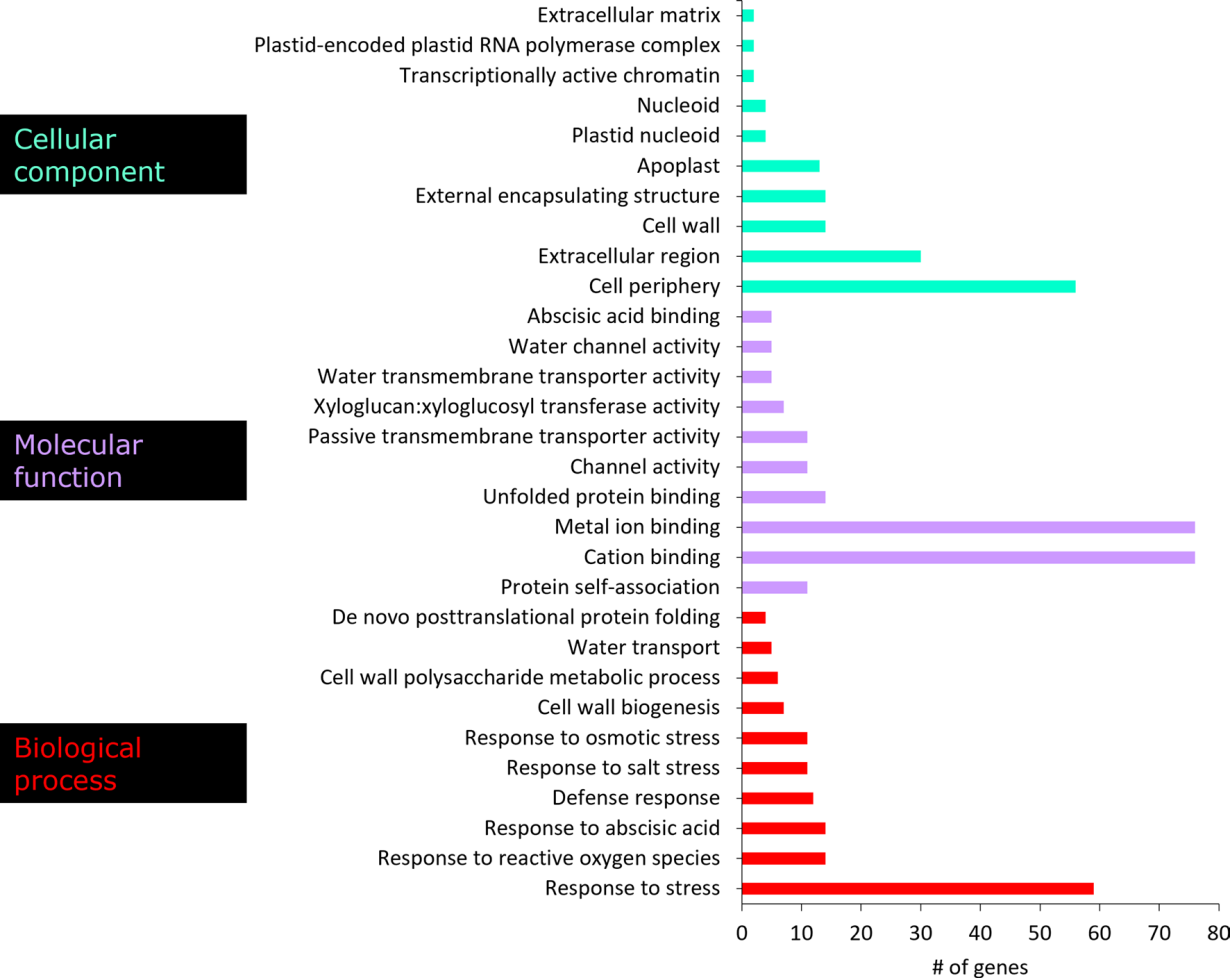
Gene ontologies for the top 10 enriched factors for Cellular Component (CC), Molecular Function (MF), and Biological Process (BP).

**Figure 3:**
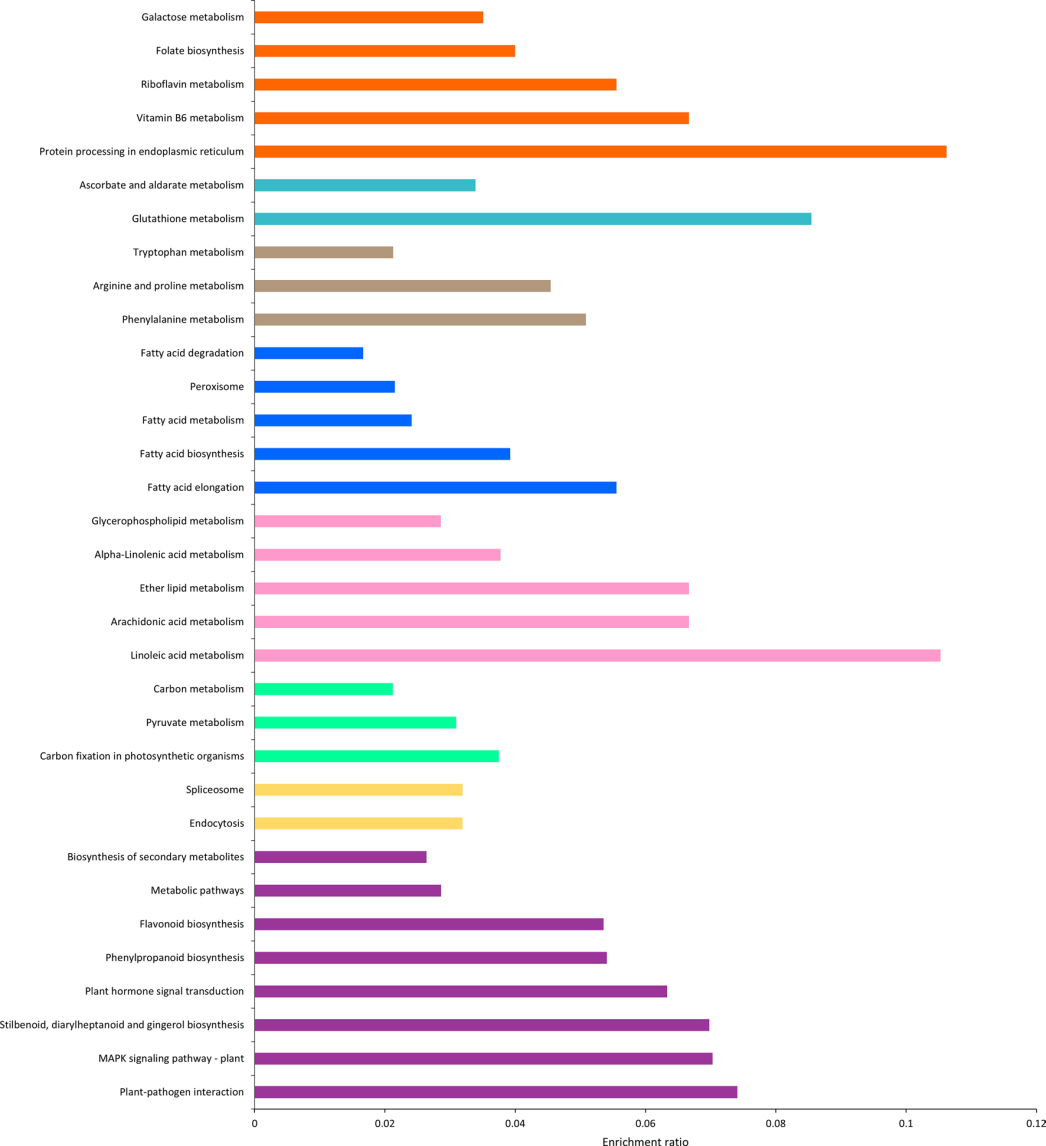
Significantly different enriched KEGG pathways in AN-SWE treated tomato plants compared to control tomato plants. The enriched ratio refers to the ratio of observed differentially expressed genes vs. the total number of genes in this KEGG pathway category for the tomato—*Solanum lycopersicum* reference genome where all genes had a corrected p-value of < 0.05. The picture was created using KOBAS 2.0.

**Figure 4:**
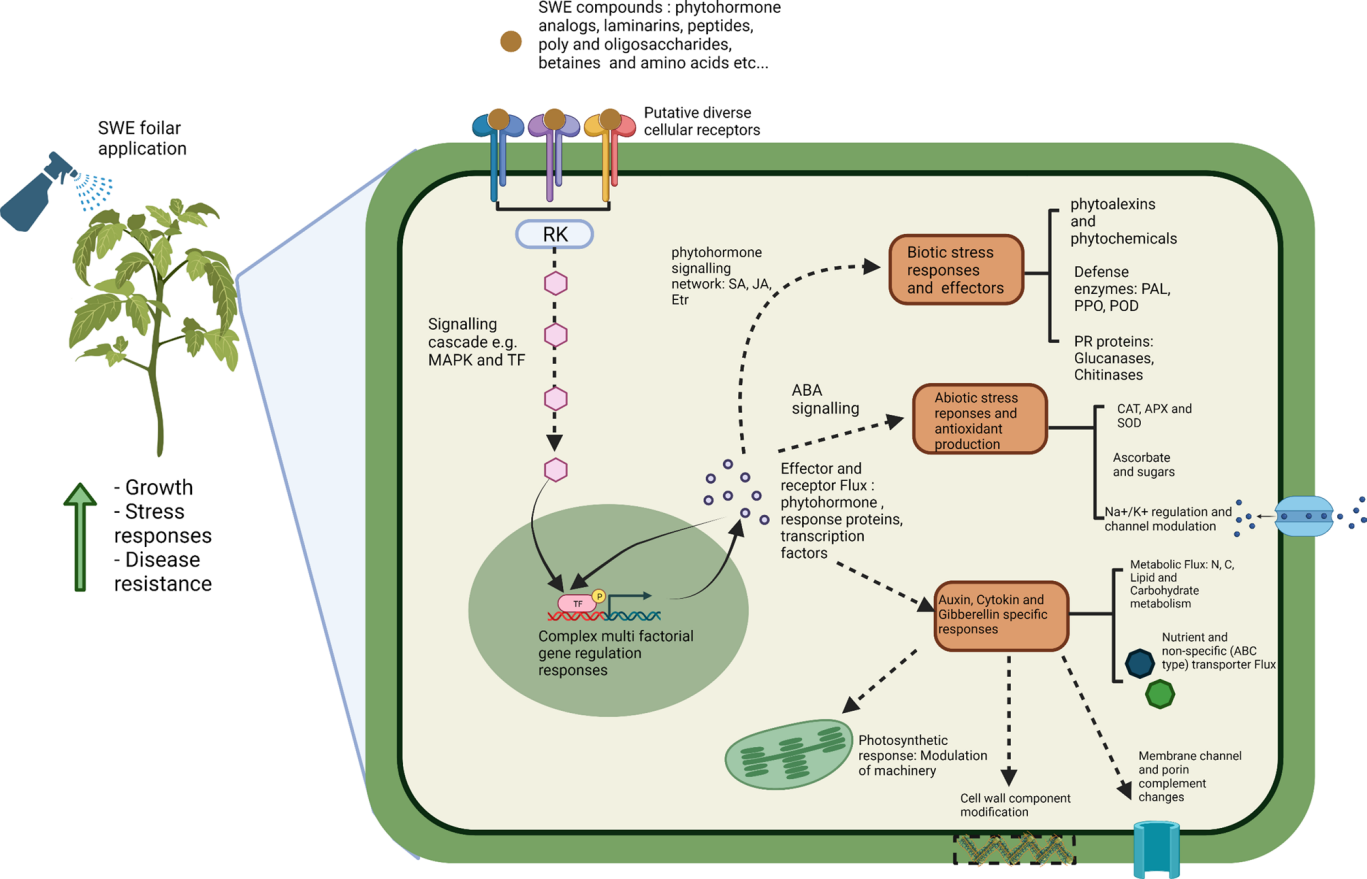
Visual representation of the key impacts of AN-SWE on the tomato transcriptome. The picture was created with BioRender.com.

**Figure 5:**
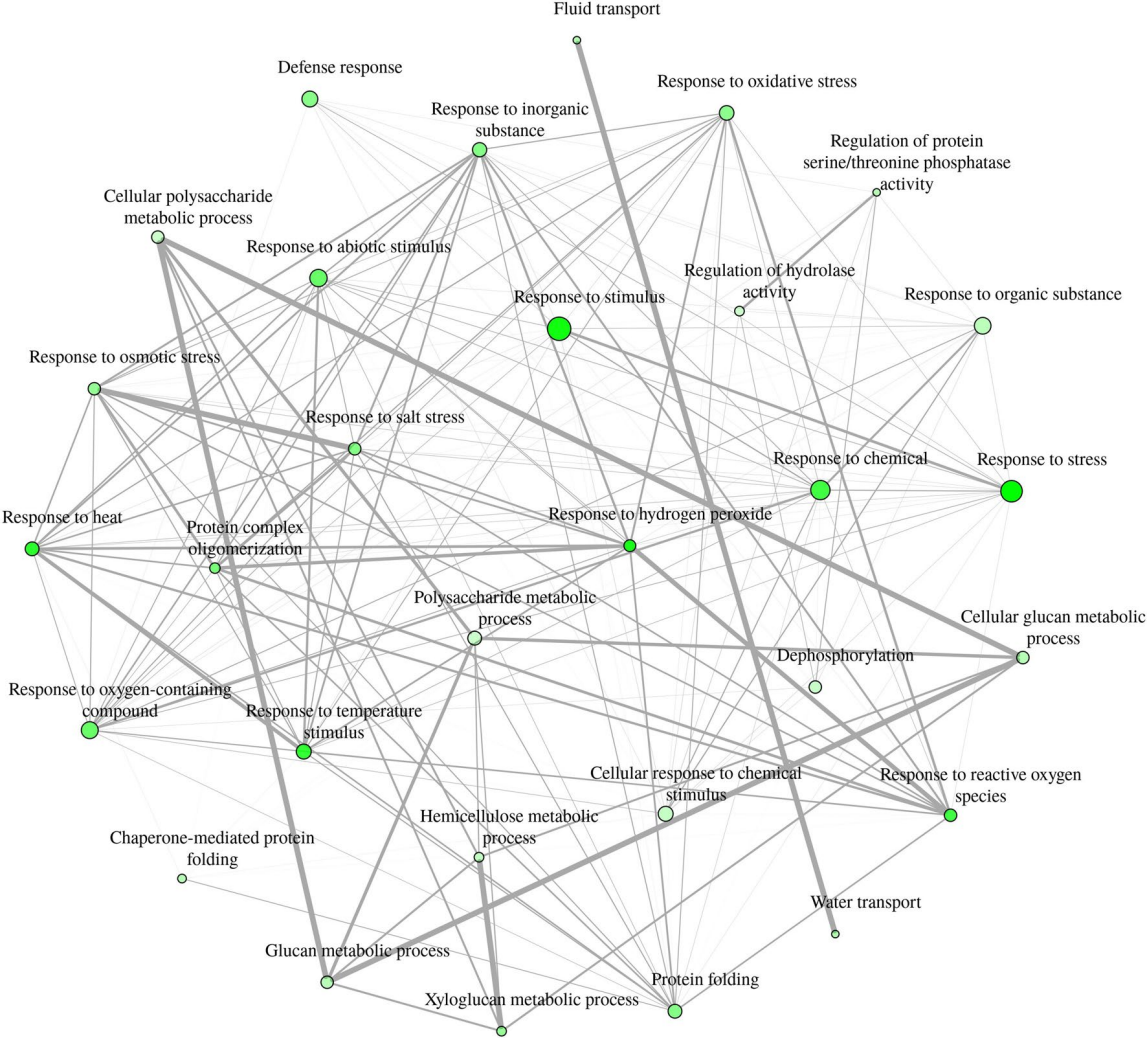
Interactive plot showing the relationship between enriched pathways. Two pathways (nodes) are connected if they share 20% or more genes. Darker nodes are more significantly enriched gene sets. Bigger nodes represent larger gene sets. Thicker edges represent more overlapped genes.

Gene ontology and pathway enrichment analysis was done to determine important groups and pathways that contained significantly expressed genes in the AN-SWE treated tomato plants. The three main ontology categories were used, i.e., biological processes (BP), cellular components (CC) and the molecular function (MF). Figure 2 illustrates these important categorical ontologies wherein the BP group comprised genes categorized under “response to stress”. Others included response to ROS, response to ABA, cell wall biogenesis, cell wall polysaccharide metabolic process, water transport and de novo post-translational protein folding. For the MF category, most genes were linked to cation and metal ion-binding. Other groups included channel activity, xyloglucan:xyloglucosyl activity and ABA-binding. Finally, the CC grouped many genes to the cell periphery, extra-cellular region and the cell wall. Additionally, these genes were then assessed for significant enrichment to various KEGG pathways. Some of these included phenylalanine metabolism, carbon fixation, secondary metabolite metabolism, MAPK-signalling, plant hormone signal transduction, glutathione metabolism, phenylpropanoid metabolism, plant-pathogen interactions and stilbenoid, diarylheptanoid and gingerol metabolism (Figs. 2 and 3). The Supplementary Table S1 highlights the genes responsible for some of the important pathways with their respective fold changes. Supplementary Table S1 lists the transcripts significantly enriched to metabolic pathways and Supplementary Table S3 presents those enriched gene ontologies with significant differences. The Supplementary Fig. S3 depicts the hierarchical clustering tree summarizing the correlation amongst significantly different pathways compared to control plants listed in the enrichment. The tree shows pathways with many shared genes that are clustered together. Figure 4 depicts the visual representation of key impacts of AS-SWE on the plant transcriptome. Figure 5 presents the interactive plot showing the relationship between enriched pathways which evidenced relationships between certain key pathways. The complexity of this dynamic plot emphasizes major convergence in molecular factors/processes encompassing key functions affecting plant growth and defense responses. There are strong indications witnessing the interrelationship between enriched pathways in plants-treated with the AN-SWE, establishing its inducer function.

### qPCR validation of DEGs using correlation analysis

**Figure 6:**
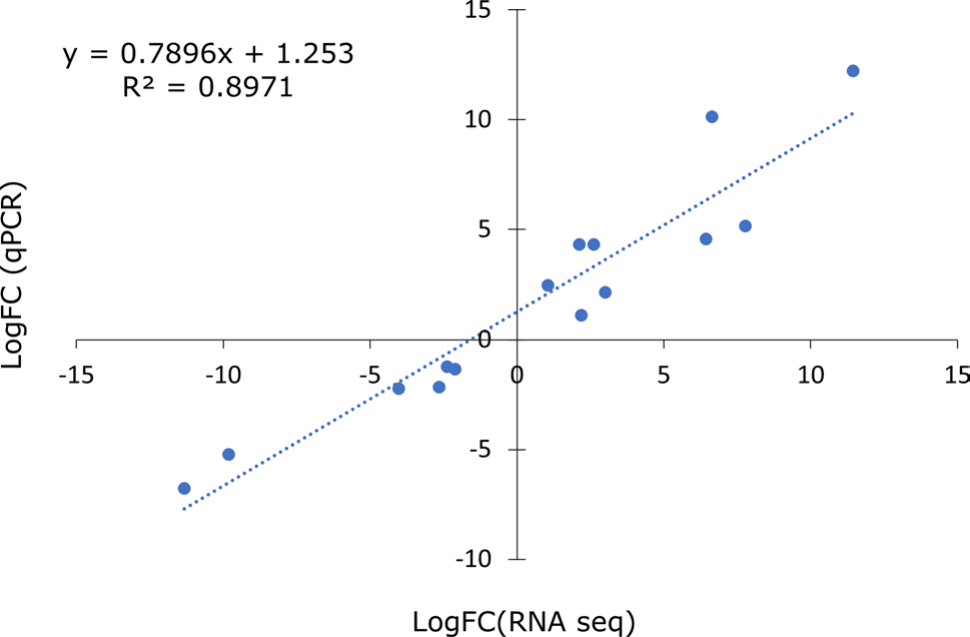
Correlation analysis of selected DEGs compared to the FC values from the qPCR independent assay with the representative R^2^ value.

Fifteen candidate genes were selected to undergo independent qPCR validation with actin as the internal house-keeping gene. Genes were selected based on their importance in plant growth and defence responses. qPCR was performed using primers with an amplification efficiency ranging between 1.97 and 1.99. Overall, there was a strong correlation between RNA sequencing results and qPCR results as indicated by an R2 value of about 89% (Fig. 6).

### Effects of AN-SWE on tomato defence enzyme content

**Figure 7:**
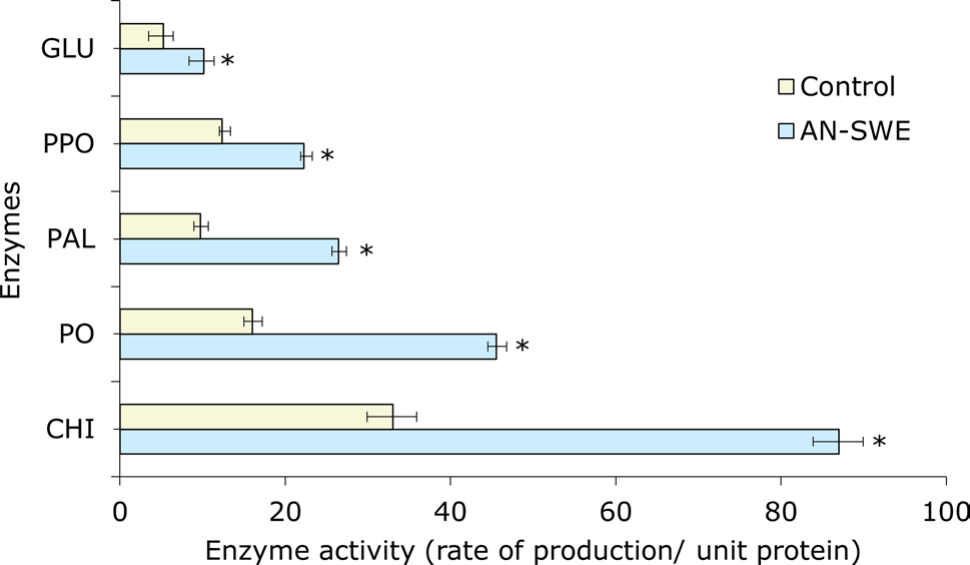
Effect of treatment with AN-SWE on plant defence enzyme activities in tomato plants. β-1,3 glucanase (GLU), peroxidase (POD), phenylalanine ammonia lyase (PAL), chitinase (CHI) and polyphenol oxidase (PPO). The data represents the mean of three plants per treatment that were analysed for significance by the Student’s t-test. Horizontal bars signify one standard deviation and “*” represents significantly different (p < 0.05) values of AN-SWE treated plants, compared to control plants.

The activities of defence enzymes include β-1,3 glucanase (GLU), peroxidase (POD), phenylalanine ammonia lyase (PAL), chitinase (CHI) and polyphenol oxidase (PPO) were quantified for AN-SWE treated plants and controls after 72 h of foliar spraying. Tomato plants treated with AN-SWE had significantly higher activities (p < 0.05) of all enzymes assayed according to the Student’s t-test (Fig. 7). Chitinase activity was the highest whereas β-1,3 glucanase was the lowest. The activity of the defence enzymes was highly correlated to the transcript levels of associated genes from transcriptomic data.

### Effect of AN-SWE on phytohormone levels in tomato

**Figure 8:**
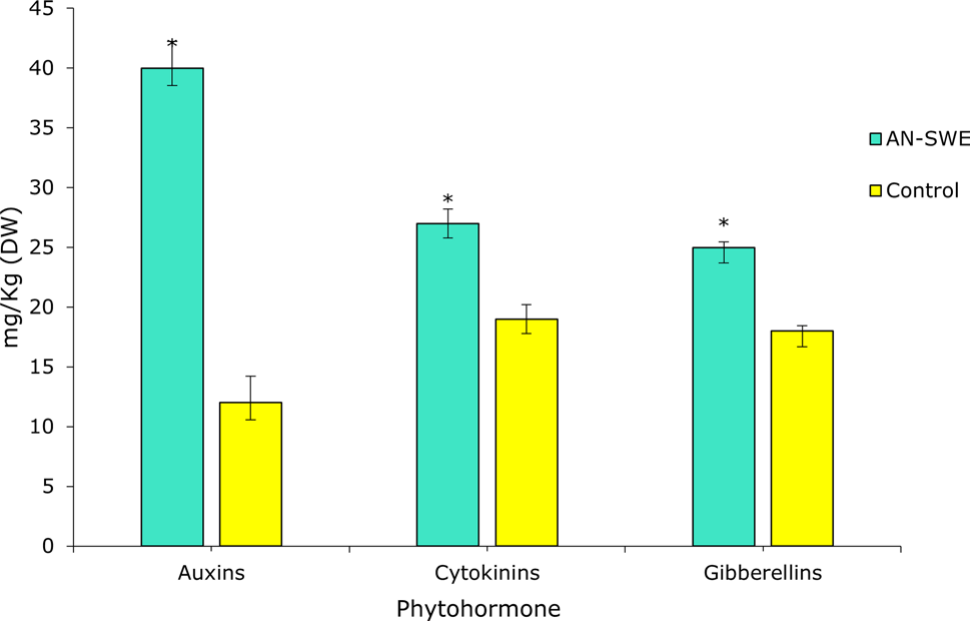
Effect of AN-SWE in tomato plants on phytohormone (auxin, cytokinin and gibberellin) levels in leaf tissue (DW). The data represents the mean of duplicate plants per treatment ± SD and was analysed for significance using the Student’s t-test where p < 0.05.

Concurrent with the transcriptomic assay, a phytohormonal panel was done to quantify some of the major plant hormones in the AN-SWE treated and control plants. Treatment with AN-SWE led to significantly increased levels of auxins, cytokinins and gibberellins (p < 0.05) according to Student’s t-test (Fig. 8). Overall, the auxin level was the highest (40 mg/kg-DW), accumulated in AN-SWE tomato plants which were followed by cytokinins (27 mg/kg) and gibberellins (25 mg/kg-DW). In AN-SWE treated plants, the levels of betaines, strigolactones, and brassinosteroids were 0.019 ± 0.00, 0.008 ± 0.00 and < 0.002 mg/kg respectively. These values were not significantly (p > 0.05) different compared to control (0.011, 0.005 and < 0.002 mg/kg respectively). Overall, brassinosteroids were the lowest of the six hormones quantified in the experiment.

### Effect of *A. nodosum* extract (Stimplex) on plant growth and micro/macro nutrient profiles

Table 1 shows the overall growth parameters of AN-SWE treated plants compared to the controls. Chlorophyll content, plant height, root length and overall dry weight were significantly higher as compared to the control (p < 0.05). Table 2 shows the micro- and macro-nutrient profile panel of both leaf and root tissue in AN-SWE treated and control plants. Calcium, phosphorous, boron, copper, iron, manganese and zinc were all significantly higher (p < 0.05) in leaves of AN-SWE-treated tomato plants compared to their controls. Similarly, the levels of calcium, potassium, boron, iron, manganese, and zinc were significantly (p < 0.05) higher in the stems of treated plants. Calcium, magnesium, potassium, sodium, boron, copper, iron, manganese and zinc were also significantly higher (p < 0.05) in roots of AN-SWE-treated tomato, as compared to the control. Overall, calcium was the most significantly accumulated macronutrient in all tissues analysed including leaves, stems and roots of treated plants, which had increased by 91.9%, 93.2% and 102.5% respectively, compared to control plants. Iron was at the highest levels in AN-SWE treated plants in leaves, stems and roots with an increase of 67.9%, 73.5% and 28.7%, respectively compared to the control plants.

**Table 1:** Effects of AN-SWE on important plant growth parameters in tomato.

Parameters	Control	AN-SWE
Chlorophyll (SPAD ratio units)	48.22 ± 0.78	58.54 ± 0.71*
Plant height (cm)	98.14 ± 1.09	128.44 ± 1.11*
Root length (cm)	9.78 ± 0.43	19.78 ± 0.38*
Total dry weight (g)	11.32 ± 1.56	23.44 ± 1.69*

The data represents the average measurements from 30 randomly selected plants ± SD. The data were analysed for significant differences by Student’s t-test and significances represented by an “*”.

**Table 2:** Effects of AN-SWE on the nutrient contents of leaves, stems and roots of tomato plants.

	AN-SWE	Control	AN-SWE	Control	AN-SWE	Control
	Leaves		Stems		Roots	
**Macronutrients**						
Calcium (%)	2.13*	1.11	1.99*	1.03	2.39*	1.18
Magnesium (%)	0.53	0.51	0.3	0.4	0.73*	0.31
Potassium (%)	3.6	3.62	2.88*	1.11	4.56*	1.13
Sodium (%)	0.03	0.04	0.03	0.03	0.09*	0.04
Nitrogen (%)	5.64	5.11	4.08	3.72	5.21	5.23
Phosphorus (%)	0.41*	0.21	0.35	0.3	0.48	0.45
Sulfur (%)	0.06	0.07	0.06	0.06	0.06	0.07
**Micronutrients**						
Boron (ppm)	24.90*	15	25.11*	16.12	44.08*	26.1
Copper (ppm)	47.08*	42.1	22.6	20.1	90.03*	62.7
Iron (ppm)	398.56*	237.3	255.13*	147.03	1157.12*	899.03
Manganese (ppm)	52.34*	27.11	36.32*	20.33	76.11*	57.2
Zinc (ppm)	47.44*	23.32	59.54*	38.22	99.22*	74.11

The data represents the average measurements from three randomly selected plants (SD values were < 0.02). The data was analysed for significant differences by Student’s t-test and significance represented by an “*”.

## Discussion

Seaweed biostimulant usage in agriculture is one strategy to boost plant yields through the enhancement of various metabolic processes which also, in turn, promotes resistance to abiotic and biotic stresses^13^. A succinct review by Rouphael and Colla provides a thorough description of these substances, including seaweed extracts, and considerations on their definitions^17^. Previous studies have confirmed that seaweed extracts are neither directly antimicrobial, nor do they contain high level of nutrients, but instead act indirectly on plants’ metabolism by triggering signalling cascades that initiate responses which lead to alleviation of various stresses and, consequently, enhanced growth and performance^3,11,14^. There is only a limited understanding of the complex mechanisms of extracts of seaweeds on plant growth processes due to their diverse array of bioactive components^15,16^. RNA sequencing gives a precise snapshot of the transcriptional changes induced by extracts of seaweeds in plants, both in time and space, which has been used in the current study. This study showed the positive effects of the AN-SWE (Stimplex) on influencing the significant expression of groups of genes responsible for multiple key metabolic processes in primed tomato plants. The key metabolic pathways included phenylalanine metabolism, carbon fixation, photosynthesis, secondary metabolite metabolism, MAPK-signalling, plant hormone signal-transduction, glutathione metabolism, phenylpropanoid metabolism, nutrient transporters, stilbenoid, diarylheptanoid and gingerol metabolism. The impacts on the plant’s transcriptome could be in part responsible for the increased plant growth paraments observed in the study including plant height, root length, plant biomass and chlorophyll content, since transcription of genes responsible for these variables were significantly influenced in treated plants compared to control. The enhanced root growth should have also improved the absorptive function of roots leading to an increase in absorption of dissolved nutrients. This was evidenced by the increased levels of nutrients partitioned in different parts of the AN-SWE treated tomato plants.

Plant cell walls are a complex natural array of structural matrices and polymers with a highly dynamic compositional relationship to both external and internal stimuli. Cell walls play an important role in supporting plant growth, development, and resistance to abiotic and biotic stresses. As the first line of defence of these sessile organisms, alterations in multiple cell wall biogenesis pathways is a useful priming mechanism against multiple stress factors including host–pathogen interactions^17^. Stimplex-treated plants had up-regulation of cell-wall biosynthesis-related genes compared to water-treated control plants. The induction of these genes validates a major priming effect of SWE treatment. Some of these genes included MYB transcription factors which are involved in mechanical protection and support via secondary cell wall biosynthesis in tomato^18^.

Further to the intense prime triggered modulation of physical defenses provided by plant cell walls, are the increased diversity and localization of phytopathogen-plant recognition receptors (PRR’s) in the plasma membrane^19^. The activation of PRRs upon detection of PAMPs/MAMPs are a major secondary front in the foundation of plant defence. Indeed, the activation of PAMP-triggered immunity in these AN-SWE-treated plants against pathogens was revealed by the up-regulation of receptor-like kinases (RLKs) as seen in the current study. This was also documented by another study which demonstrated that the extracts of southern oceans’ kelp *Durvillaea potatorum* and the temperate, northern hemisphere, *A. nodosum (*Seasol*)*-treated *Arabidopsis* plants were able to resist *Phytophthora cinnamomi* infections through up-regulation of this basal defence from RLKs as well as a plethora of other defense mechanisms^20^.

Salicylic acid (SA), jasmonic acid (JA) and ethylene (ET) play important roles in pathogen defence mechanisms in plants. Biotrophic pathogens are targeted by the SA-signalling route, which initiates programmed cell death, whereas necrotrophic pathogens are deterred by JA and ET signalling networks^21^. In the current study, genes responsible for these defence-mediated signalling pathways were up-regulated including *PinII* (jasmonic acid) *Etr1* (ethylene), *PR-1a* (salicylic acid). This was further confirmed by qPCR studies. Auxins, gibberellins and ethylene are major phytohormones, well-known for plant growth and developmental regulation which are also recognized as critical controllers of plant defence responses^23^. In the current study, auxin-signalling genes such as auxin-responsive protein SAUR21-like and indole-3-acetic acid-amido synthetase were highly up-regulated in AN-SWE-treated tomato tissues. Similarly, the ethylene responsive element binding protein (*EREB*) (543,712) and gibberellin 20-oxidase 4(*GA20ox4*) (100,191,112) were also up-regulated in AN-SWE primed plants. These genes have also been linked to several defence signalling networks in tomato^22^. The elevated levels of hormonal compounds in the AN-SWE treated plants further confirmed the enhanced synthesis and activity of phytohormones which should have a strong correlation and contribution to plant growth and productivity. Apart from the induced synthesis of plant growth hormones, algal extracts such as the AN-SWE are known to contain low levels of cytokinins, gibberellins, auxins, abscisic acid, and betaines^23^. These would have also impacted the regulation of corresponding target genes which would have in turn contributed to improved plant growth and function^5^.

The work by Goni et al. using microarrays also reported some similar responses in *Arabidopsis thaliana* upon treatment with a proprietary extract of *A. nodosum*^2^. An ethyl acetate fraction of *A. nodosum* was also shown to up-regulate several stress responsive genes through microarray analysis in *A. thaliana* under NaCl-induced salt stress thereby promoting salinity tolerance^24^. An extract prepared from *A. nodosum* was also able to up-regulate various metabolic pathways in *Brassica napus*^25^. Furthermore, a recent study using *A. nodosum* seaweed extract, SuperFifty (SF-BioAtlantis) by Omidbakhshfard et al. showed that the extract was able to up-regulate marker genes responsible for the prevention of oxidative stress in *A. thaliana* during water stress growing conditions^26^. The current study used a commercial *A. nodosum* extract which is different from previous studies, and with tomato as a model. Though the gene regulation trends observed in the previous studies were somewhat like those noticed here, the current study provided a detailed and in-depth perspective and evidence into the gene expression profile due to the nature of the techniques used and analysis conducted.

Calcium-dependent protein kinases (CDPKs) are essential players in plant signalling that phosphorylate a variety of substrates such as transcription factors, ion channels, and metabolic enzymes, to convert calcium signals into physiological responses. CDPKs play critical roles in shoot and root establishment, pollen tube formation, stomatal motions, hormone signalling, transcriptional remodelling, and stress resistance due to their wide range of targets. In the current study, two CDPKs were up-regulated, and this coupled with significant increases in calcium levels in tomato tissues treated with AN-SWE demonstrated a probable signalling cascade event taking place to promote biotic stress resistance. WRKY transcription factors were also up-regulated in tomato plants treated with AN-SWE for example the WRKY transcription factor 13 which was up-regulated in tomato under drought and salt stress in another study^27,28^. This therefore reiterates the multifaceted mechanism of action of AN-SWE, not only in aiding with biotic stress tolerance but in alleviating abiotic stress as well. A garnesyl pyrophosphate synthase (sly:101258792) was also up-regulated which is directly involved in the synthesis of sterols and sesquiterpenes. These two compounds have been documented to show^29,30^ plant growth and defence regulatory mechanisms.

The up-regulation of secondary metabolic synthesis genes was also recorded in the current study. KEGG-enrichment showed that phenylpropanoid biosynthesis was significantly enhanced with AN-SWE treatment. Plant phenolic levels were also significantly higher in AN-SWE-treated tomato plants. This increase in phenols would also account for the significant increase in PAL through activation of the shikimate pathway wherein erythrose 4-phosphate is coupled with phosphoenolpyruvate (PEP) to produce phenylalanine and subsequently the production of phenolic chemicals^31^. Furthermore, several peroxidases (peroxidase 3, 11 & 12) were also up-regulated in the current study. Peroxidases have been linked to oxidative burst events which occur in order to promote disease resistance^32^. This again coupled with the significant increase in calcium levels in AN-SWE-treated plants gives strong confidence in promoting the defence-priming mechanism in which calcium is linked with defence-signalling^32^. Additionally, peroxidases have also been linked to a wide range of physiological activities, including the production of lignin and suberin, cell wall cross-linking, and phytoalexin production, as well as the metabolism of ROS and reactive nitrogen species (RNS). Furthermore, peroxidase enzymatic activity in AN-SWE*-*treated tomato plants were significantly higher than in the control plants. All these would contribute to elevated levels of resistance to pathogen infection. Increases in peroxidase accumulation have been linked to increased resistance to bacterial wilt, bacterial spot and early blight in tomato plants^33,34^. Another group of up-regulated secondary metabolite genes were phospholipases (e.g., phospholipase A1-II and phospholipase D). The activation of phospholipases is responsible for the creation of key defence-signalling molecules such as oxylipins and jasmonates, as well as the powerful secondary messenger phosphatidic acid, which has been demonstrated to influence the activity of several defence-signalling proteins^35^. This group has also been linked with several growth related signalling (e.g., cytokinin, auxin and gibberellin) mechanisms as was documented in tomato^36^ and *Arabidopsis*^37^.

There was an overall trend of significantly expressed genes involved in water and nutrient transporters, ion channel activators, ion binding proteins and membrane transporters in AN-SWE-treated tomato plants in this study. The increase in activity of these genes and proteins would have contributed to the overall improvement in plants’ mechanisms including enhanced water absorptive capacity, and mobilization of nutrients and elements. This has been corroborated by enhanced levels of nutrient elements in plant parts as seen in the present study.

As with many transcriptomic studies, due to limitations of reference gene coverage and functional profiling, many differentially expressed genes remained unannotated. The metabolic role of these genes represents an under-utilized genetic resource in understanding the complex impact of biostimulants and in expanding the limited marker gene profile used in qPCR-based studies. Such targets might represent unique elements involved in plant defence pathways, pattern recognition receptors (PRRs) which are not fully characterised in plants and mitigating factors reducing the metabolic burden of defence pathway up-regulation^38–40^. Further understanding can be achieved using comparative phenotype and gene expression studies utilizing *Arabidopsis* mutants of key defence and metabolic pathways^40^. Docking studies can also be employed utilizing tertiary and secondary protein and RNA structures of hypothetical proteins from this present study to determine their role, if any, in interactions which lead to the key primed AN-SWE molecular events documented here. Additionally, the AN-SWE components can also be separated and assessed either singly or in combination to fully understand the comprehensive and synergistic relationships that exist with each fraction.

This study altogether showed that AN-SWE-treated tomato plants caused a favourable pattern of regulation of genes involved in the synthesis of key metabolites which acted as activators of signal transduction contributing to diverse functions and leading to multiple effects. The summary of transcriptomic changes and effects in plants induced by the seaweed extract is presented in Fig. 4. The activated processes were multiple and complex which would have led to enhanced plant defence, plant growth and productivity, photosynthetic activity control, resilience to abiotic stresses, and improved tissue repair. These responses allowed the plants to have better adaptability to growing conditions which are in constant flux. This study, therefore, provides gene-based evidence for the positive effects observed by application of this commercial *Ascophyllum* extract, Stimplex and supports the intensive use of this product as a biological input to achieve sustainability in crop production.

## Methods

### Experimental system

Healthy tomato (Hybrid-61) seedlings (6-weeks old) were transplanted into 1L plastic planters filled with soil and peat moss (1:1) and watered daily using drip-tip irrigation under greenhouse conditions (i.e., 30 °C, 70–85% relative humidity, and 600–1000 μmol photons/m^2^/s^1^ with ~ 12 h photoperiod). Plants were subsequently left to acclimatize for 1 week after which the *A. nodosum* seaweed extract (AN-SWE) was applied. The *A. nodosum* extract (Stimplex, EPA reg.# 75287-3) was obtained from Acadian SeaPlants Ltd. (Dartmouth, NS, Canada) which has a chemical profile as follows: cytokinin as kinetin 0.01%/ 100 ppm activity, protein/amino acids 3–6%, lipid 1%, alginic acid 12–18%, fucose-containing polymers 12–15%, mannitol 5–6%, other carbohydrates 10–20%. The treatment consisted of a foliar application of 0.5% AN-SWE v/v (TDS-680 ppm) up to saturation and in the same way, water was sprayed to plants as controls. The spray was carefully applied as a fine mist to the shoots with no dripping on the soil. Two randomly selected plants each with the AN-SWE treatment and water controls were selected 72 h after foliar application. There were 40 plants maintained per treatment and a plastic barrier was laid between treatments to prevent cross-contamination. Leaves were sampled at the 3rd and 4th nodes from the base for RNA extraction and sequencing.

### RNA extraction and sequencing

Total RNA was extracted from tomato leaves (500 mg) utilizing the Trizol reagent (Invitrogen) according to the manufacturer’s protocol. The isolated RNA was then quantified, and the quality was assessed using a Jenway Genova NanoSpec and de-naturing agarose gel electrophoresis. RNA samples were then sent to a commercial sequencing provider (Novogene, California, USA) for RNA sequencing. cDNA library preparation was done using the Illumina RNA NEBNext Ultra ll kit and sequencing was done using the NovaSeq 6000 platform. Paired-end (150 bp) sequencing was done with approximately 20 million reads per sample. The experiment's raw RNA sequencing fastq data files were deposited in the NCBI Short Read Archive (SRA) database under the bio-project PRJNA753600.

### Bioinformatic analysis

The Galaxy on-line bioinformatic analysis services (www.usegalaxy.eu and www.usegalaxy.org) were used to obtain differentially expressed genes (DEGs) using a reference-based transcriptomic approach. The bio-informatic pipeline used is depicted in Supplementary Fig. S1. FastQC^41^ Version 0.72 was used to check the quality of raw reads followed by Trimmomatic^42^ Version 0.38.1 to trim low-quality leading and trailing bases (QC < 30) and remove short reads (< 100 bp). Subsequently, the reads were reanalysed with FastQC to confirm adequate filtering and that only high-quality reads were retained. HISAT2^43^ Version 2.1.0 was then used to map the reads to the *Solanum lycopersicum*-Cultivar: Heinz 1706 (GCF_000188115.4) reference genome. FeatureCounts^44^ was used to obtain raw counts using the annotation file obtained from NCBI which was manually edited for the tool’s requirements. EdgeR^45^ Version 3.24.1 was then utilized to obtain DEGs utilizing the trimmed mean of mean (TMM) values factor of normalization^46^. The raw differentially expressed gene (DEG) file obtained was then subjected to filtering with a false discovery rate (FDR) < 0.05 and a p-value < 0.05. Transcripts fitting these two parameters were considered significantly differentially expressed. Additionally, a log-fold change (LogFC) of ≤ − 2 or ≥ + 2 was used to delineate downregulated and up-regulated transcripts^47^.

### Gene ontology and pathway enrichment

Gene ontology analysis was done using Blast2Go^48^ and ShinyGo^49^ on all DEGs. Terms belonging to the three main ontological groups (i.e., Biological processes, BP; Molecular function, MF; and Cellular component, CC) were considered as significantly enriched when the corrected p-value and FDR were both ≤ 0.05 according to the Benjamini–Hochberg method^50^. Blast2Go and KOBAS 2.0^51^ were used to determine significantly enriched pathways using a FDR corrected p-value of ≤ 0.05.

### Validation of DEGs using qPCR

The Quantitative polymerase chain reaction method was used for validating the results of the RNA-seq analysis utilizing three biological replicates per treatment. From the DEGs list, fifteen important genes that were linked to defence and growth hormonal signalling pathways that were typically up-regulated and down-regulated were chosen for the qPCR assay. Primers were designed using the Integrated DNA Technologies-Primer Quest tool and the oligo primers were synthesized by IDT (Iowa, USA). The primer sequences were found in Table S1. The actin gene (LOC101250165) was used as house-keeping gene control. Correlation plots were made using the RNA-seq Log FC against the qPCR log FC.

### Effects of AN-SWE on tomato defence enzyme content

Treated tomato plants were sampled (72 h) in triplicate for defence enzyme quantitation. Enzymes quantified included β-1,3 glucanase (GLU), peroxidase (POD), phenylalanine ammonia lyase (PAL), chitinase (CHI) and polyphenol oxidase (PPO). GLU was quantified using absorbance 500 nm and the amount of released glucose (umol/h/ml) with laminarin as the substrate^52^. POD was quantified using the substrate pyrogallol and measured at 420 nm (increased absorbance/min/mg)^53^. PAL was measured by absorbance changes at 290 nm from the conversion of l-phenylalanine to trans-cinnamic acid (nmol/min/g)^54^. CHI was quantified by the rate of production of *N*-acetylglucosamine (μg/g/h) wherein chitin (crab shells) was utilized as the substrate and the absorbance was measured at 585 nm^52^. PPO content was determined using catechol as substrate and the absorbance was measured at 495 nm (change in absorbance/min/g)^55^.

### Tomato phyto-hormone analysis

Plant samples from the same experimental trial were used for the phyto-hormone analysis. Leaves collected from three AN-SWE foliar sprayed and control tomato plants were sent to the J.H.G. Analytical Services Limited (Waterford, Ireland) for endogenous plant hormone quantification. The quantification of hormones included betaines, auxins, cytokinins, gibberellins strigolactones, and brassinosteroids. The content of betaines, auxins and cytokinins were quantified using high-performance liquid chromatography–photodiode array detection (HPLC–PDA). The content of gibberellins, strigolactones, and brassinosteroids were quantified using Gas chromatography–mass spectrometry (GC–MS).

### Effect of AN-SWE on plant growth and nutrient content

The treated plants from the trial were used for plant growth and nutrient content analysis. After five applications of the treatments, up to the reproductive plant growth stage, 20 randomly selected treated, as well as control, plants were measured for plant height, chlorophyll content (atLEAF+, FT Green LLC, Detroit, USA) and dry weight biomass. The shoots and roots from three randomly selected plants were dried and samples were sent for micro and macro nutritional profiling at Agro Services International (Orange City, FL, USA).

### Methodology

All methods were carried out in accordance with relevant guidelines and regulations.

## Supplementary Information

Supplementary Information.

## Data Availability

The RNA sequencing fastq data files were deposited in the NCBI (SRA) database under the bio-project PRJNA753600. All the sequence analysis data generated during this study were included in this published article along with Supplementary information files.
