# Supplementary material for: Transcriptomic changes induced by applications of a commercial extract of Ascophyllum nodosum on tomato plants
Source: Sci Rep. 2022 May 16;12:8042. doi: 10.1038/s41598-022-11263-z (PMC9110418; doi:10.1038/s41598-022-11263-z)
Supplement: Supplementary file 1 — Supplementary Information. [file 41598_2022_11263_MOESM1_ESM.docx]

**Supplementary Information**

**Transcriptomic changes induced by applications of a commercial extract of *Ascophyllum nodosum* on tomato plants**

Omar Ali, Adesh Ramsubhag, Stephen Daniram Benn Jr. Ramnarine and Jayaraj Jayaraman*

Department of Life Sciences, Faculty of Science and Technology, The University of the West Indies, St. Augustine, Trinidad and Tobago.

*Corresponding author: [jayauwi@gmail.com](mailto:jayauwi@gmail.com)

Quality Assessment

**FastQC**

**Trimmomatic**


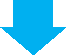


Mapping

**HISAT2**


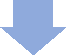


Counting

**featureCounts**


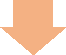


Differential Expression

**edgeR**


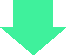


Gene Ontology & Enrichment Pathway Enrichment Graphical Plots

**Blast2GO**

**KOBAS**

**ShinyGO**

Figure S1. RNA-sequencing analysis pipeline.


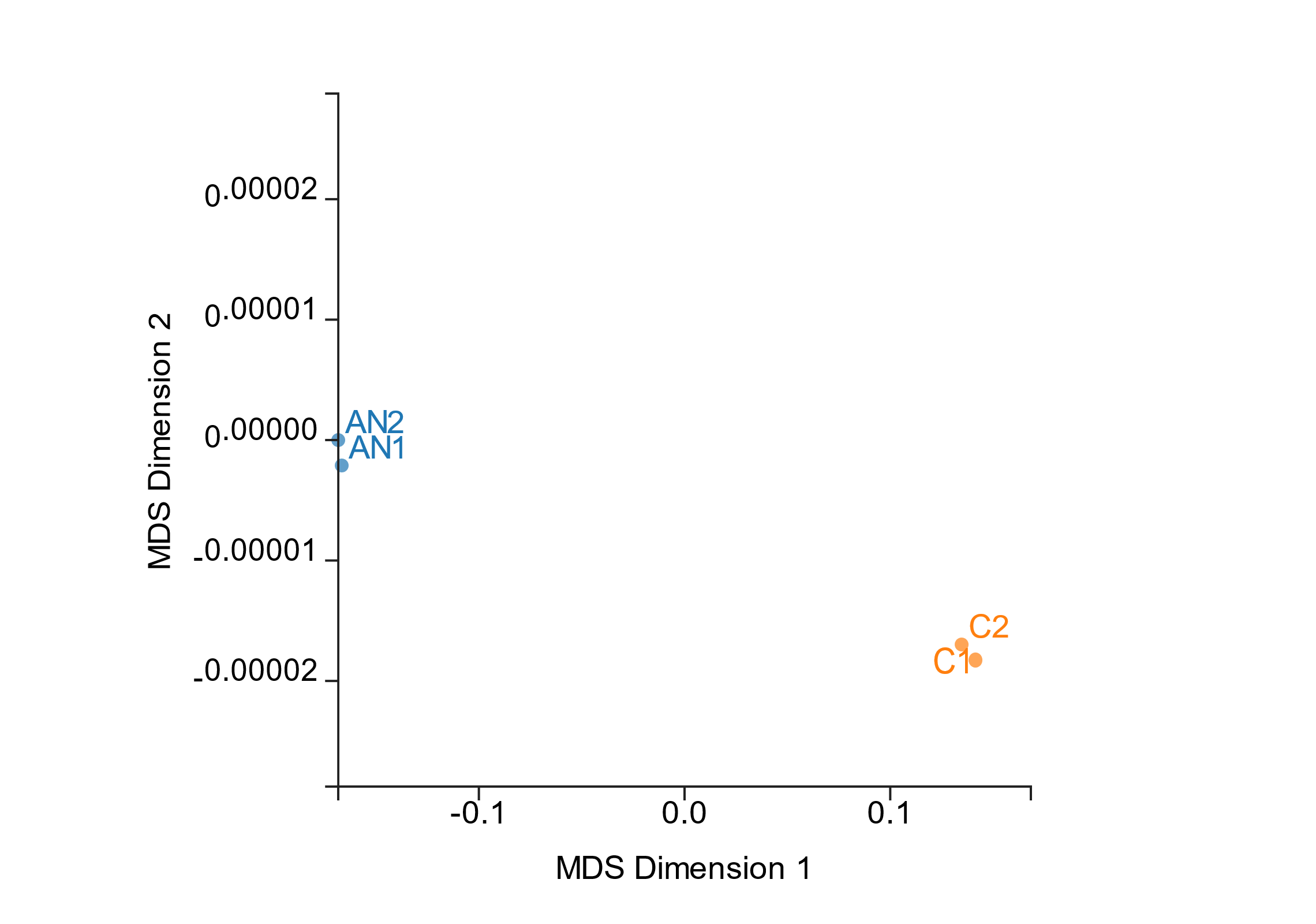

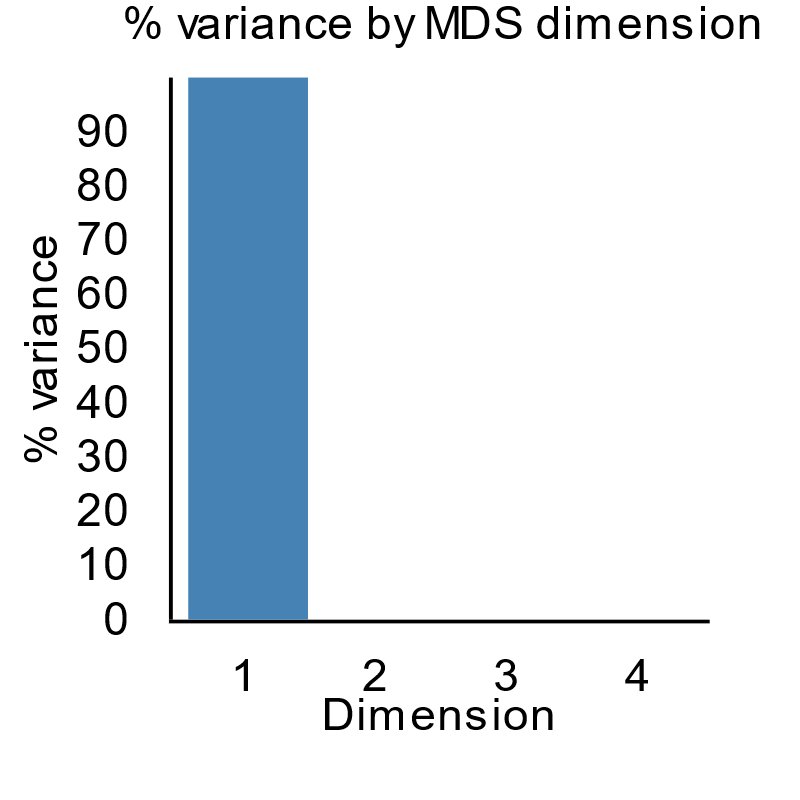


Figure S2. MDS plot highlighting variances with each treatment replication.


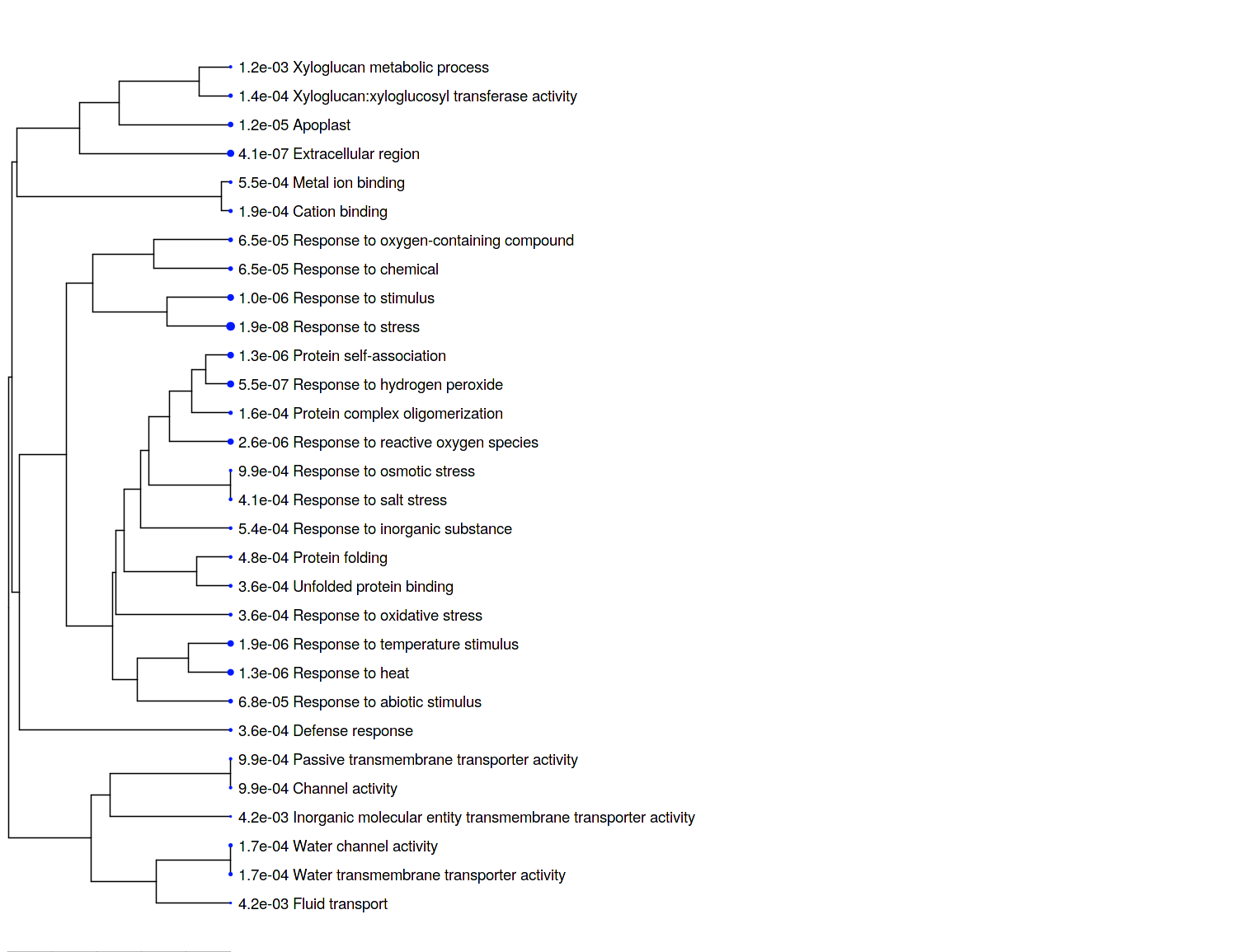


Figure S3. A hierarchical clustering tree summarizing the correlation among significant pathways listed in the enrichment. Pathways with many shared genes are clustered together. The larger dots indicate more significant P-values.

Table S1. Primers used for qPCR validation

| **Entrez ID** | **Gene name** | **Forward Primer (5’ – 3’)** | **Reverse Primer (5’ – 3’)** | **Amplicon size (bp)** |
| --- | --- | --- | --- | --- |
| 101249032 | pathogen-related protein | GCTGGTGAGAAGGAAACAACTA | TGTCATAAATTGGAGGGCTACC | 121 |
| 101246961 | wound-induced proteinase inhibitor | GGCAAAGCAGGTTTGAAAGAG | GAACTGTGACAGAGCCAACA | 137 |
| 101251231 | ethylene-responsive transcription factor | AGAACTCAGCTTGGTGTTAAGT | GCCTTCTGCTTGTGTCTCTAT | 113 |
| 100191112 | gibberellin 20-oxidase | CAATGCCACTCGTCTTGTTAATG | GTGGCTTCCCAAAGAAGAAATC | 128 |
| 101262738 | probable indole-3-acetic acid-amido synthetase | GATGTGGTAGAAGTGGATGGTT | CCGCTACTAATTGGAGGTCTTT | 124 |
| 101249857 | Vicilin | GGTAGAACACAGGGACCTTTC | GTCCCTCAATTGCTCGTATCT | 108 |
| 101249641 | glyceraldehyde-3-phosphate dehydrogenase | TGACCACGGTCCATTCTATTAC | CCACCTCTCCAGTCTTTCATC | 147 |
| 101248335 | ferredoxin | GAGGAAGAACTCACAAGCTGAA | CGAACGAGCGACAAACAAATC | 144 |
| 101256331 | cysteine synthase | TTCACTCCTCTCATTCACTTTCTT | ACTAACCTTTCCAGGCGTAATC | 117 |
| 101254231 | laccase-17 | TCGATGCTGACGGAGTTTAC | CATAAGGTCGAGCAGTCATGTA | 138 |
| 101258963 | abscisic acid receptor | CTGCCACACTAGCGGTTAAA | ATGGGTGTGGTGATGCAATA | 104 |
| 544276 | isocitrate lyase | GAAGCTATGGAGGACACTCAAG | CACTGCCAGCCAGATACATAA | 132 |
| 101248599 | histidine decarboxylase | CCTCTCTCCTCACTCTCTCAATA | GGGAGGGAGGTGAATTGTATATG | 133 |
| 543619 | xyloglucan endotransglycosylase | AGCCCACTTGAGAGTGATATTG | CATAACTTGTGTCCCTCGATACTT | 113 |
| 101259193 | adenylate isopentenyltransferase 3 | CAACCCTGATGGCGATTACA | AAGCATCTTGTCCCGAGTATTT | 121 |
| 101250165 | Actin | TTCACGCAGGCTGGATTT | TTTGACCCATCCCTACCATAAC | 103 |

Table S2. Enriched KEGG pathway terms

| Pathway Term | logFC | Gene ID | Name |
| --- | --- | --- | --- |
| Plant hormone signal transduction | -3.82 | 101250944 | abscisic acid receptor PYL4 (LOC101250944) |
|  | 2.54 | 544204 | AREB-like protein (AREB) |
|  | 3.33 | 101243847 | auxin-induced protein 15A-like (LOC101243847) |
|  | -3.48 | 543712 | ethylene responsive element binding protein (EREB) |
|  | 4.15 | 101262506 | histidine kinase 4 (LOC101262506) |
|  | 2.31 | 101253938 | histidine-containing phosphotransfer protein 1 (LOC101253938) |
|  | -2.77 | 101248225 | histidine-containing phosphotransfer protein 4-like (LOC101248225) |
|  | 3.24 | 101249794 | probable protein phosphatase 2C 51 (LOC101249794) |
|  | 2.52 | 100316895 | protein phosphatase 2C AHG3 homolog (PP2C-2) |
|  | -2.02 | 101247936 | protein TIFY 10A-like (LOC101247936) |
|  | 2.72 | 101248058 | two-component response regulator ARR8 (LOC101248058) |
|  | 2.40 | 101245668 | xyloglucan endotransglucosylase/hydrolase protein 24-like (LOC101245668) |
|  | 7.77 | 100191112 | gibberellin 20-oxidase 4 (GA20ox4) |
|  | 2.61 | 101262738 | probable indole-3-acetic acid-amido synthetase GH3.5 (LOC101262738) |
|  | 1.04 | 101259193 | adenylate isopentenyltransferase 3 |
| Plant Pathogen Interaction | -2.86 | 101253387 | calcium-binding allergen Ole e 8-like (LOC101253387) |
|  | -2.02 | 101260188 | calcium-dependent protein kinase 32-like (LOC101260188) |
|  | 2.54 | 101244669 | cyclic nucleotide-gated ion channel 1-like (LOC101244669) |
|  | 3.33 | 543900 | EIX receptor 1 (Eix1) |
|  | 3.32 | 101264183 | heat shock protein 83 (LOC101264183) |
|  | 2.29 | 101260143 | heat shock protein 90 (HSP90) |
|  | 4.15 | 101245247 | probable calcium-binding protein CML44 (LOC101245247) |
|  | 2.31 | 104645862 | probable LRR receptor-like serine/threonine-protein kinase At3g47570 (LOC104645862) |
|  | -3.26 | 101245784 | probable WRKY transcription factor 29 (LOC101245784) |
|  | -2.77 | 544042 | Pti5 (LOC544042) |
|  | 3.24 | 101267202 | putative calcium-binding protein CML19 (LOC101267202) |
|  | 2.52 | 101249560 | RPM1-interacting protein 4 (LOC101249560) |
|  | 2.18 | 101249032 | pathogenesis-related protein (LOC101249032) |
|  | 2.12 | 101246961 | wound-induced proteinase inhibitor 1 (LOC101246961) |
|  | 6.64 | 101251231 | ethylene-responsive transcription factor WRI1 (LOC101251231) |
| Glutathione metabolism | -3.26 | 101250402 | glutathione S-transferase U17-like (LOC101250402) |
|  | -2.02 | 543628 | glutathione S-transferase/peroxidase (BI-GST/GPX) |
|  | 2.58 | 101258987 | L-ascorbate peroxidase 2, cytosolic (LOC101258987) |
|  | -3.26 | 101263597 | leucine aminopeptidase 1, chloroplastic-like (LOC101263597) |
|  | 8.06 | 101266149 | probable glutathione S-transferase (LOC101266149) |
| Phenylpropanoid biosynthesis | -2.68 | 101259903 | 8-hydroxygeraniol dehydrogenase-like (LOC101259903) |
|  | -2.05 | 101253556 | agmatine coumaroyltransferase-2-like (LOC101253556) |
|  | 3.86 | 101257526 | beta-glucosidase 18 (LOC101257526) |
|  | 2.31 | 101262487 | feruloyl CoA ortho-hydroxylase 2-like (LOC101262487) |
|  | -2.15 | 101248973 | peroxidase 11 (LOC101248973) |
|  | -3.39 | 101250523 | peroxidase 3 (LOC101250523) |
|  | -3.20 | 101253636 | peroxidase 43-like (LOC101253636) |
|  | -2.06 | 101260035 | putative Peroxidase 48 (LOC101260035) |
|  | 3.51 | 104648161 | salutaridinol 7-O-acetyltransferase-like (LOC104648161) |
|  | 3.62 | 104648251 | salutaridinol 7-O-acetyltransferase-like (LOC104648251) |
|  | -2.30 | 544103 | scopoletin glucosyltransferase (twi1) |
|  | 2.73 | 101256448 | tetrahydrocannabinolic acid synthase-like (LOC101256448) |
| MAPK signaling pathway - plant | -2.02 | 101250944 | abscisic acid receptor PYL4 (LOC101250944) |
|  | -2.63 | 101251136 | endochitinase (LOC101251136) |
|  | -2.78 | 543712 | ethylene responsive element binding protein (EREB) |
|  | 3.03 | 101264683 | mitogen-activated protein kinase kinase NPK1-like (LOC101264683) |
|  | 2.31 | 104645862 | probable LRR receptor-like serine/threonine-protein kinase At3g47570 (LOC104645862) |
|  | 3.19 | 101249794 | probable protein phosphatase 2C 51 (LOC101249794) |
|  | -3.26 | 101245784 | probable WRKY transcription factor 29 (LOC101245784) |
|  | 4.08 | 104647127 | protein MKS1-like (LOC104647127) |
|  | -2.04 | 100316895 | protein phosphatase 2C AHG3 homolog (PP2C-2) |
| Transporters | 2.44 | 101264389 | potassium channel SKOR-like (LOC101264389) |
|  | 3.83 | 101265010 | sulfate transporter 3.4 (LOC101265010) |
|  | 6.96 | 101264742 | ABC transporter G family member 10 (LOC101264742) |
|  | 3.35 | 101260863 | boron transporter 1 (LOC101260863) |
|  | 8.94 | 101250514 | aquaporin TIP3-2 (LOC101250514) |
|  | 2.27 | 1444424174 | amino acid transporter AVT6C-like (LOC101266946), transcript variant X1, mRNA |
|  | -3.01 | 101246876 | ammonium transporter 2 (LOC101246876) |
|  | -2.28 | 101263195 | bidirectional sugar transporter SWEET10-like (LOC101263195) |

Table S3. Transcripts significantly enriched in seaweed treated tomato plants

| **logFC** | **Gene ID** | **Name** |
| --- | --- | --- |
| -2.50 | 544052 | 1-aminocyclopropane-1-carboxylate oxidase 1 (ACO1) |
| 2.69 | 101259906 | 2-succinylbenzoate--CoA ligase, chloroplastic/peroxisomal (LOC101259906) |
| 6.01 | 101260109 | 3-ketoacyl-CoA synthase 3-like (LOC101260109) |
| -2.27 | 543602 | 8-hydroxygeraniol dehydrogenase (LOC543602) |
| -2.68 | 101259903 | 8-hydroxygeraniol dehydrogenase-like (LOC101259903) |
| -6.48 | 101265715 | acetate/butyrate--CoA ligase AAE7, peroxisomal (LOC101265715) |
| 3.96 | 101257483 | acidic mammalian chitinase-like (LOC101257483) |
| -2.05 | 101253556 | agmatine coumaroyltransferase-2-like (LOC101253556) |
| 2.72 | 101251942 | alpha-1,4-glucan-protein synthase [UDP-forming] 1-like (LOC101251942) |
| -2.37 | 101254788 | alpha-amylase (LOC101254788) |
| 2.48 | 101266758 | amidophosphoribosyltransferase, chloroplastic-like (LOC101266758) |
| -3.05 | 101250072 | apyrase-like (LOC101250072) |
| -2.05 | 101264167 | ATP synthase subunit beta, mitochondrial (LOC101264167) |
| 3.86 | 101257526 | beta-glucosidase 18 (LOC101257526) |
| 2.85 | 101256086 | chitinase 6 (LOC101256086) |
| 5.42 | 101256331 | cysteine synthase-like (LOC101256331) |
| -2.12 | 101264898 | cytochrome c oxidase subunit 6a, mitochondrial-like (LOC101264898) |
| 7.77 | 101262353 | cytochrome P450 86A8-like (LOC101262353) |
| -2.09 | 543721 | DNA (cytosine-5)-methyltransferase (MET) |
| -2.63 | 544125 | endo-1,4-beta-D-glucanase(cel7) |
| -2.63 | 101251136 | endochitinase (LOC101251136) |
| -2.12 | 101245995 | endoglucanase 24-like (LOC101245995) |
| 6.43 | 101258792 | farnesyl pyrophosphate synthase 1, mitochondrial (LOC101258792) |
| 3.10 | 101262300 | galactinol--sucrose galactosyltransferase-like (LOC101262300) |
| 3.17 | 778310 | geranylgeranyl pyrophosphate synthase 1 (GGPS1) |
| 3.89 | 100191112 | gibberellin 20-oxidase 4 (GA20ox4) |
| -2.79 | 544211 | gibberellin 20-oxidase-2 (20ox-2) |
| 4.53 | 101259235 | glutamate decarboxylase 4(LOC101259235) |
| -3.26 | 101250402 | glutathione S-transferase U17-like (LOC101250402) |
| -2.02 | 543628 | glutathione S-transferase/peroxidase (BI-GST/GPX) |
| -2.03 | 101249641 | glyceraldehyde-3-phosphate dehydrogenase, cytosolic (LOC101249641) |
| 2.47 | 101249664 | histidine decarboxylase (LOC101249664) |
| 3.59 | 101248599 | histidine decarboxylase-like (LOC101248599) |
| 2.11 | 101259946 | inorganic pyrophosphatase 1-like (psi14C) |
| 2.01 | 101254427 | inositol oxygenase 1-like (LOC101254427) |
| 2.78 | 544276 | isocitrate lyase (LOC544276) |
| 3.29 | 101250644 | lactosylceramide 4-alpha-galactosyltransferase-like(LOC101250644) |
| 2.58 | 101258987 | L-ascorbate peroxidase 2, cytosolic (LOC101258987) |
| -3.26 | 101263597 | leucine aminopeptidase 1, chloroplastic-like (LOC101263597) |
| 2.50 | 101246288 | long chain acyl-CoA synthetase 4-like (LOC101246288) |
| 3.94 | 101255777 | NADP-dependent malic enzyme, chloroplastic (LOC101255777) |
| -2.15 | 101248973 | peroxidase 11(LOC101248973) |
| -3.39 | 101250523 | peroxidase 3(LOC101250523) |
| -3.20 | 101253636 | peroxidase 43-like (LOC101253636) |
| -11.33 | 101245149 | phosphoenolpyruvate carboxylase 4 (LOC101245149) |
| -3.32 | 101245304 | phospholipase D p1 (LOC101245304) |
| -3.33 | 101265207 | polygalacturonase-like (LOC101265207) |
| 8.06 | 101266149 | probable glutathione S-transferase (LOC101266149) |
| 3.22 | 101266750 | probable glutathione S-transferase (LOC101266750) |
| 2.72 | 101267339 | probable glutathione S-transferase (LOC101267339) |
| -2.07 | 101267638 | probable glutathione S-transferase (LOC101267638) |
| 2.36 | 101245157 | probable isoaspartyl peptidase/L-asparaginase 2(LOC101245157) |
| 2.01 | 101257960 | probable pectate lyase 3 (LOC101257960) |
| -2.61 | 778293 | probable pectate lyase P18(9612) |
| -4.45 | 101264170 | probable polyamine oxidase 4 (LOC101264170) |
| -2.61 | 101248050 | probable polygalacturonase At1g80170 (LOC101248050) |
| 2.92 | 101247598 | probable trans-2-enoyl-CoA reductase, mitochondrial (LOC101247598) |
| -2.34 | 101268445 | proline dehydrogenase 2, mitochondrial-like (LOC101268445) |
| 2.42 | 101248079 | protochlorophyllide reductase-like (LOC101248079) |
| -4.53 | 101249252 | purple acid phosphatase 17-like (LOC101249252) |
| -6.81 | 101255659 | putative amidase C869.01 (LOC101255659) |
| -2.06 | 101260035 | putative Peroxidase 48 (LOC101260035) |
| 3.51 | 104648161 | salutaridinol 7-O-acetyltransferase-like (LOC104648161) |
| 3.62 | 104648251 | salutaridinol 7-O-acetyltransferase-like (LOC104648251) |
| 4.22 | 101263843 | stachyose synthase (LOC101263843) |
| 2.73 | 101256448 | tetrahydrocannabinolic acid synthase-like (LOC101256448) |
| 7.21 | 101255254 | triacylglycerol lipase SDP1-like (LOC101255254) |
| 2.95 | 101247013 | type I inositol 1,4,5-trisphosphate 5-phosphatase 11(LOC101247013) |
| -2.01 | 101259914 | uncharacterized LOC101259914(LOC101259914) |
| -2.31 | 543813 | uncharacterized LOC543813(LOC543813) |
| -3.23 | 543814 | uncharacterized LOC543814(LOC543814) |

Table S4. List of significantly enriched ontologies

| **Enrichment FDR** | **Genes in list** | **Total genes** | **Functional Category** |
| --- | --- | --- | --- |
| 2.13E-11 | 41 | 784 | Response to chemical |
| 2.13E-11 | 74 | 2184 | Response to stimulus |
| 2.13E-11 | 29 | 390 | Response to oxygen-containing compound |
| 3.11E-11 | 19 | 149 | Response to temperature stimulus |
| 1.65E-10 | 15 | 89 | Response to heat |
| 6.49E-10 | 47 | 1142 | Response to stress |
| 2.31E-09 | 28 | 454 | Response to abiotic stimulus |
| 3.01E-09 | 8 | 17 | Regulation of protein serine/threonine phosphatase |
| 2.73E-08 | 24 | 379 | Cellular response to chemical stimulus |
| 2.73E-08 | 21 | 284 | Cellular response to organic substance |
| 2.73E-08 | 8 | 22 | Abscisic acid binding |
| 3.79E-08 | 8 | 23 | Alcohol binding |
| 5.19E-08 | 8 | 24 | Isoprenoid binding |
| 5.62E-08 | 23 | 371 | Extracellular region |
| 5.62E-08 | 96 | 3979 | Integral component of membrane |
| 5.62E-08 | 97 | 4040 | Intrinsic component of membrane |
| 5.62E-08 | 44 | 1211 | Cell periphery |
| 5.62E-08 | 8 | 25 | Protein phosphatase inhibitor activity |
| 5.62E-08 | 8 | 25 | Hormone binding |
| 7.00E-08 | 8 | 26 | Phosphatase inhibitor activity |
| 1.09E-07 | 28 | 564 | Response to organic substance |
| 1.47E-07 | 13 | 111 | Unfolded protein binding |
| 1.62E-07 | 8 | 29 | Response to hydrogen peroxide |
| 2.09E-07 | 8 | 30 | Regulation of phosphoprotein phosphatase activity |
| 2.39E-07 | 15 | 164 | Protein folding |
| 2.57E-07 | 8 | 31 | Regulation of protein dephosphorylation |
| 3.27E-07 | 8 | 32 | Regulation of phosphatase activity |
| 4.36E-07 | 9 | 47 | Protein phosphatase regulator activity |
| 4.86E-07 | 10 | 64 | Response to reactive oxygen species |
| 4.96E-07 | 8 | 34 | Regulation of dephosphorylation |
| 5.80E-07 | 9 | 49 | Phosphatase regulator activity |
| 7.82E-07 | 7 | 24 | Protein self-association |
| 1.09E-06 | 24 | 484 | Response to hormone |
| 1.43E-06 | 15 | 192 | Response to inorganic substance |
| 1.43E-06 | 78 | 3229 | Biological regulation |
| 1.43E-06 | 24 | 492 | Response to endogenous stimulus |
| 1.59E-06 | 8 | 40 | Monocarboxylic acid binding |
| 2.32E-06 | 10 | 77 | Abscisic acid-activated signaling pathway |
| 2.45E-06 | 16 | 231 | Hormone-mediated signaling pathway |
| 2.69E-06 | 10 | 79 | Regulation of hydrolase activity |
| 2.69E-06 | 10 | 79 | Cellular response to abscisic acid stimulus |
| 2.69E-06 | 10 | 79 | Cellular response to alcohol |
| 2.85E-06 | 13 | 150 | Response to alcohol |
| 2.85E-06 | 13 | 150 | Response to abscisic acid |
| 3.91E-06 | 7 | 31 | Chaperone-mediated protein folding |
| 3.95E-06 | 16 | 242 | Cellular response to hormone stimulus |
| 5.90E-06 | 7 | 33 | Protein complex oligomerization |
| 5.90E-06 | 16 | 250 | Cellular response to endogenous stimulus |
| 8.68E-06 | 6 | 22 | Water transmembrane transporter activity |
| 8.68E-06 | 6 | 22 | Water channel activity |
| 8.68E-06 | 14 | 196 | Enzyme inhibitor activity |
| 9.59E-06 | 14 | 198 | Response to lipid |
| 9.59E-06 | 11 | 116 | Cellular response to lipid |
| 1.00E-05 | 33 | 958 | Plasma membrane |
| 1.17E-05 | 70 | 2958 | Regulation of biological process |
| 1.37E-05 | 19 | 375 | Enzyme regulator activity |
| 1.39E-05 | 10 | 97 | Carboxylic acid binding |
| 1.39E-05 | 10 | 97 | Organic acid binding |
| 1.55E-05 | 13 | 178 | Regulation of protein modification process |
| 1.62E-05 | 23 | 538 | Inorganic molecular transmembrane transporter activity |
| 1.89E-05 | 12 | 153 | Channel activity |
| 1.89E-05 | 12 | 153 | Passive transmembrane transporter activity |
| 2.80E-05 | 12 | 159 | Molecular transducer activity |
| 2.88E-05 | 10 | 106 | Protein processing in endoplasmic reticulum |
| 3.04E-05 | 15 | 255 | Regulation of molecular function |
| 3.04E-05 | 38 | 1265 | Molecular function regulator |
| 3.04E-05 | 13 | 191 | Cellular response to oxygen-containing compound |
| 4.15E-05 | 33 | 1033 | Transmembrane transport |
| 4.50E-05 | 11 | 139 | Plant hormone signal transduction |
| 4.52E-05 | 6 | 30 | *de novo* protein folding |
| 4.96E-05 | 15 | 267 | Regulation of cellular protein metabolic process |
| 5.31E-05 | 58 | 2399 | Cation binding |
| 6.75E-05 | 57 | 2361 | Metal ion binding |
| 6.81E-05 | 9 | 93 | Anion transmembrane transport |
| 6.81E-05 | 4 | 9 | Protein histidine kinase binding |
| 6.81E-05 | 9 | 93 | Response to salt stress |
| 7.17E-05 | 15 | 277 | Regulation of protein metabolic process |
| 7.22E-05 | 17 | 351 | Defense response |
| 8.76E-05 | 14 | 247 | Regulation of catalytic activity |
| 9.50E-05 | 35 | 1184 | Cellular response to stimulus |
| 9.84E-05 | 61 | 2634 | Nucleus |
| 0.000103743 | 62 | 2702 | Regulation of cellular process |
| 0.000103743 | 13 | 218 | Cell wall |
| 0.000103743 | 13 | 218 | External encapsulating structure |
| 0.000103743 | 4 | 10 | Misfolded protein binding |
| 0.000123103 | 34 | 1151 | Transporter activity |
| 0.00013094 | 33 | 1104 | Transmembrane transporter activity |
| 0.000133823 | 5 | 22 | Protein refolding |
| 0.000133823 | 5 | 22 | *de novo* posttranslational protein folding |
| 0.000133823 | 5 | 22 | Chaperone cofactor-dependent protein refolding |
| 0.000148599 | 11 | 162 | Regulation of phosphate metabolic process |
| 0.000148599 | 11 | 162 | Regulation of phosphorus metabolic process |
| 0.000150599 | 9 | 105 | Response to osmotic stress |
| 0.000209783 | 4 | 12 | Protein folding chaperone |
| 0.000215372 | 9 | 110 | Apoplast |
| 0.000300459 | 5 | 26 | Heat shock protein binding |
| 0.000356743 | 26 | 812 | Cell communication |
| 0.000362084 | 24 | 717 | Signal transduction |
| 0.000386892 | 24 | 721 | Signaling |
| 0.000386892 | 4 | 14 | Fluid transport |
| 0.000386892 | 4 | 14 | Water transport |
| 0.000512115 | 4 | 15 | Cellular response to unfolded protein |
| 0.000512115 | 4 | 15 | Response to unfolded protein |
| 0.000736151 | 6 | 51 | Amino acid transmembrane transport |
| 0.000788031 | 10 | 163 | Protein dephosphorylation |
| 0.000788031 | 5 | 32 | Xyloglucan:xyloglucosyl transferase activity |
| 0.000850521 | 48 | 2074 | Localization |
| 0.000895247 | 5 | 33 | Xyloglucan metabolic process |
| 0.001052127 | 3 | 7 | Histidine phosphotransfer kinase activity |
| 0.001302924 | 6 | 57 | Organic acid transmembrane transport |
| 0.001302924 | 6 | 57 | Carboxylic acid transmembrane transport |
| 0.001555363 | 6 | 59 | Protein kinase binding |
| 0.001555363 | 6 | 59 | Amino acid transport |
| 0.001821888 | 11 | 218 | Dephosphorylation |
| 0.001948268 | 14 | 339 | Cell wall organization or biogenesis |
| 0.00218874 | 6 | 63 | Kinase binding |
| 0.002201775 | 4 | 22 | Response to topologically incorrect protein |
| 0.002201775 | 4 | 22 | Cellular response to topologically incorrect protein |
| 0.002201775 | 78 | 4064 | Cytoplasm |
| 0.002201775 | 9 | 152 | Signaling receptor activity |
| 0.00256344 | 10 | 191 | Monooxygenase activity |
| 0.002568404 | 5 | 42 | Cellular response to heat |
| 0.002568404 | 31 | 1200 | Transition metal ion binding |
| 0.003152087 | 7 | 96 | Cell wall macromolecule metabolic process |
| 0.003308945 | 11 | 236 | Response to oxidative stress |
| 0.003663941 | 44 | 1981 | Establishment of localization |
| 0.003759996 | 11 | 240 | Anion transport |
| 0.003806961 | 5 | 46 | Amino acid transmembrane transporter activity |
| 0.004003192 | 3 | 11 | Cellular response to cytokinin stimulus |
| 0.004003192 | 3 | 11 | Cytokinin-activated signaling pathway |
| 0.004550467 | 5 | 48 | Response to cytokinin |
| 0.004581814 | 13 | 330 | Ion transmembrane transport |
| 0.004581814 | 11 | 247 | Cell wall organization |
| 0.005218744 | 43 | 1960 | Transport |
| 0.005432896 | 9 | 175 | Anion transmembrane transporter activity |
| 0.005606982 | 6 | 77 | Spliceosome |
| 0.005612057 | 55 | 2712 | Hydrolase activity |
| 0.005706432 | 40 | 1791 | Regulation of nitrogen compound metabolic process |
| 0.005962727 | 8 | 142 | Cell differentiation |
| 0.006269336 | 11 | 258 | External encapsulating structure organization |
| 0.00627694 | 3 | 13 | Lipid storage |
| 0.006577446 | 41 | 1867 | Regulation of cellular metabolic process |
| 0.006825682 | 40 | 1811 | Regulation of primary metabolic process |
| 0.00733026 | 10 | 223 | Lipid binding |
| 0.007642249 | 3 | 14 | Glutamine family amino acid catabolic process |
| 0.007642249 | 3 | 14 | Response to high light intensity |
| 0.008084387 | 19 | 644 | Sequence-specific DNA binding |
| 0.008141672 | 6 | 84 | Organic acid transport |
| 0.008141672 | 6 | 84 | Carboxylic acid transport |
| 0.00862747 | 7 | 117 | Plant-pathogen interaction |
| 0.00886089 | 42 | 1963 | Regulation of macromolecule metabolic process |
| 0.009094727 | 20 | 704 | Developmental process |
| 0.009365996 | 5 | 58 | Hemicellulose metabolic process |
| 0.009365996 | 43 | 2032 | Regulation of metabolic process |
| 0.009868808 | 19 | 658 | Ion transport |
| 0.009960813 | 5 | 59 | Response to cold |
| 0.011191869 | 11 | 281 | Polysaccharide metabolic process |
| 0.011191869 | 17 | 564 | Ion transmembrane transporter activity |
| 0.011232565 | 8 | 160 | Cellular developmental process |
| 0.011232565 | 5 | 61 | Organic acid transmembrane transporter activity |
| 0.011232565 | 5 | 61 | Carboxylic acid transmembrane transporter activity |
| 0.012757061 | 6 | 93 | Fruit development |
| 0.013285569 | 12 | 333 | Intracellular signal transduction |
| 0.013318055 | 6 | 94 | MAPK signaling pathway |
| 0.015120865 | 4 | 39 | Response to jasmonic acid |
| 0.015618939 | 2 | 5 | Macrolide binding |
| 0.015618939 | 2 | 5 | FK506 binding |
| 0.016355728 | 28 | 1194 | Metabolic pathways |
| 0.019533311 | 3 | 20 | Inorganic anion transmembrane transport |
| 0.021774815 | 13 | 403 | Hydrolase activity, acting on glycosyl bonds |
| 0.022106201 | 5 | 72 | Cell wall polysaccharide metabolic process |
| 0.022106201 | 7 | 141 | Cellular glucan metabolic process |
| 0.022295769 | 2 | 6 | Phloem development |
| 0.022295769 | 2 | 6 | Plastid-encoded plastid RNA polymerase complex |
| 0.024446035 | 3 | 22 | Aminoglycan catabolic process |
| 0.024446035 | 3 | 22 | Chitin metabolic process |
| 0.024446035 | 3 | 22 | Chitin catabolic process |
| 0.024446035 | 3 | 22 | Amino sugar catabolic process |
| 0.024446035 | 3 | 22 | Glucosamine-containing compound catabolic process |
| 0.024711023 | 7 | 145 | Glucan metabolic process |
| 0.02666282 | 36 | 1728 | Cellular component organization or biogenesis |
| 0.02666282 | 12 | 367 | Hydrolase activity, hydrolyzing O-glycosyl compounds |
| 0.02670753 | 23 | 953 | Organic substance catabolic process |
| 0.026993029 | 3 | 23 | Glucosamine-containing compound metabolic process |
| 0.026993029 | 4 | 47 | Nutrient reservoir activity |
| 0.028344087 | 2 | 7 | Regulation of systemic acquired resistance |
| 0.028344087 | 2 | 7 | Positive regulation of histone modification |
| 0.028344087 | 2 | 7 | Positive regulation of chromatin organization |
| 0.028344087 | 12 | 372 | ATPase activity |
| 0.028344087 | 2 | 7 | Linoleic acid metabolism |
| 0.029392992 | 3 | 24 | Aminoglycan metabolic process |
| 0.029392992 | 3 | 24 | Chitin binding |
| 0.031289351 | 6 | 115 | Oxidoreductase activity |
| 0.032578318 | 5 | 81 | Phosphorelay signal transduction system |
| 0.032578318 | 3 | 25 | Chitinase activity |
| 0.036422317 | 6 | 119 | Cell wall biogenesis |
| 0.038298673 | 32 | 1528 | Cellular component organization |
| 0.039096188 | 6 | 121 | Organic anion transport |
| 0.04050698 | 17 | 654 | Pyrophosphatase activity |
| 0.04467948 | 17 | 661 | Hydrolase activity, acting on acid anhydrides |
| 0.045159348 | 2 | 9 | Positive regulation of chromosome organization |
| 0.048115324 | 3 | 29 | Amino sugar metabolic process |
| 0.048422937 | 12 | 402 | Iron ion binding |
| 0.048422937 | 16 | 613 | Nucleoside-triphosphatase activity |
| 0.048923028 | 17 | 669 | Hydrolase activity, acting on acid anhydrides |
| 0.049513786 | 36 | 1812 | Oxidoreductase activity |
